# Ethyl 2-[1,3-dioxo-6-(piperidin-1-yl)-2,3-dihydro-1*H*-benz[*de*]isoquinolin-2-yl]acetate

**DOI:** 10.1107/S1600536811022707

**Published:** 2011-06-18

**Authors:** Song Xia, Chun-Ling Zheng, Fei-Fei He, Ya-Bin Shi, Hai-Bo Wang

**Affiliations:** a College of Food Science and Light Industry, Nanjing University of Technology, Xinmofan Road No. 5 Nanjing, Nanjing 210009, People’s Republic of China; b College of Science, Nanjing University of Technology, Xinmofan Road No. 5 Nanjing, Nanjing 210009, People’s Republic of China

## Abstract

In the title compound, C_21_H_22_N_2_O_4_, the naphthalimide unit is almost planar (r.m.s. deviation = 0.081Å). The carboximide N atom and the five C atoms of the eth­oxy­carbonyl­methyl substituent also lie close to a common plane (r.m.s. deviation = 0.119Å), which subtends an angle of 71.06 (8)° to the naphthalamide plane. The piperidine ring adopts a chair conformation. In the crystal, inter­molecular C—H⋯O hydrogen bonds link the mol­ecules into zigzag chains along the *a* axis.

## Related literature

For general background to applications of 1,8-naphthalimides, see: McAdam *et al.* (2003 ▶); Fülöp *et al.* (2009 ▶). For a related structure, see: Hanton *et al.* (2010 ▶). For bond-length data, see: Allen *et al.* (1987 ▶).
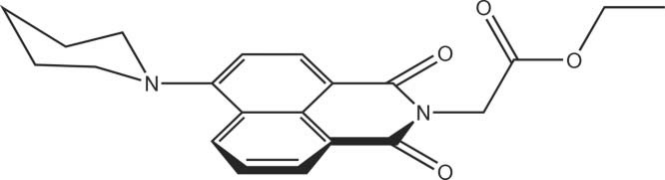


## Experimental

### 

#### Crystal data

C_21_H_22_N_2_O_4_
*M* *_r_* = 366.41
Orthorhombic, 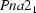

*a* = 10.959 (2) Å
*b* = 18.037 (4) Å
*c* = 9.3330 (19) Å
*V* = 1844.8 (6) Å^3^
*Z* = 4
Mo *K*α radiation
μ = 0.09 mm^−1^
*T* = 293 K
0.30 × 0.20 × 0.10 mm

#### Data collection

Enraf–Nonius CAD-4 diffractometer
Absorption correction: ψ scan (North *et al.*, 1968 ▶) *T*_min_ = 0.973, *T*_max_ = 0.991
3547 measured reflections
1808 independent reflections
1280 reflections with *I* > 2σ(*I*)
*R*_int_ = 0.039
3 standard reflections every 200 reflections  intensity decay: 1%

#### Refinement

*R*[*F* ^2^ > 2σ(*F* ^2^)] = 0.045
*wR*(*F* ^2^) = 0.115
*S* = 1.00
1808 reflections
244 parameters
2 restraints
H-atom parameters constrained
Δρ_max_ = 0.21 e Å^−3^
Δρ_min_ = −0.13 e Å^−3^

### 

Data collection: *CAD-4 EXPRESS* (Enraf–Nonius, 1994 ▶); cell refinement: *CAD-4 EXPRESS*; data reduction: *XCAD4* (Harms & Wocadlo, 1995 ▶); program(s) used to solve structure: *SHELXS97* (Sheldrick, 2008 ▶); program(s) used to refine structure: *SHELXL97* (Sheldrick, 2008 ▶); molecular graphics: *SHELXTL* (Sheldrick, 2008 ▶); software used to prepare material for publication: *PLATON* (Spek, 2009 ▶).

## Supplementary Material

Crystal structure: contains datablock(s) global, I. DOI: 10.1107/S1600536811022707/sj5150sup1.cif

Structure factors: contains datablock(s) I. DOI: 10.1107/S1600536811022707/sj5150Isup2.hkl

Supplementary material file. DOI: 10.1107/S1600536811022707/sj5150Isup3.cml

Additional supplementary materials:  crystallographic information; 3D view; checkCIF report

## Figures and Tables

**Table 1:** Hydrogen-bond geometry (Å, °)

*D*—H⋯*A*	*D*—H	H⋯*A*	*D*⋯*A*	*D*—H⋯*A*
C1—H1*A*⋯O2^i^	0.97	2.60	3.455 (6)	147
C1—H1*B*⋯O1^ii^	0.97	2.51	3.373 (5)	149
C5—H5*A*⋯O2^iii^	0.97	2.44	3.219 (6)	138
C18—H18*B*⋯O4^iv^	0.97	2.56	3.315 (5)	135

Symmetry codes: (i) 
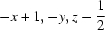
; (ii) 
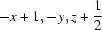
; (iii) 
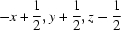
; (iv) 
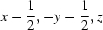
.

**Fig. 1.**
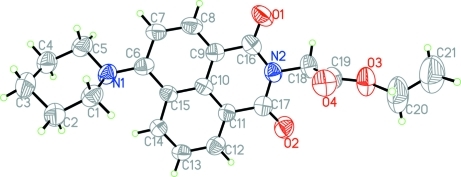
The molecular structure of the title molecule, with the atom-numbering scheme. Displacement ellipsoids are drawn at the 50% probability level.

**Fig. 2.**
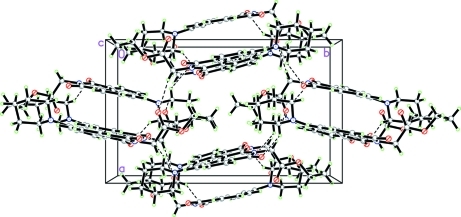
A packing diagram of the title compound viewed down the c axis. Dashed lines indicate intermolecular C-H···O interactions.

## supplementary crystallographic information

### Comment

1,8-naphthalimide derivatives are recognized to have an importance in dye and medicinal chemistry. They can be used as intermediates in the synthesis of  organic pigments, in biological fluorescent labeling and as optical brighteners, pH-dependent sensors, laser and electroluminscent dyes and liquid crystals (McAdam *et al.*, 2003). We have selected 4-substituted 1,8-naphthalimides to use in the synthesis of fluorophore groups, since they are highly photostable, cheap and their chemical modification is straightforward. Moreover, these dyes exhibit large Stoke's shifts due to the formation of an intramolecular charge transfer (ICT) state upon absorption of light. (Fülöp *et al.* 2009).

We report here the crystal structure of the title compound, N-[(2-Ethoxy)-2-oxo-ethyl]-4-piperidino-1,8-naphthalimide. In the structure of the title compound (Fig. 1), the bond lengths (Allen *et al.*, 1987) and angles are within normal ranges (Hanton *et al.*, 2010). In the crystal structure, intermolecular C-H···O hydrogen bonds link the molecules into zig-zag chains along the *a* axis, to form a stable structure (Fig. 2).

### Experimental

The title compound, 1H-Benz[de]isoquinoline- 2(3H)-acetic acid, 6-(piperidin-1-yl)-1,3-dioxo-, ethyl ester was prepared by a method similar to that reported in the literature (Fülöp *et al.* 2009). 1H-Benz [de]isoquinoline- 2(3H)-acetic acid, 6-bromo-1,3-dioxo-, ethyl ester(3.82 g, 10.5 mmol) was dissolved in N-methylpyrrolidone (NMP, 58.5 mL) and piperidine (4.5 mL, 52.5 mmol) together with triethylamine (TEA, 14.8 mL, 105 mmol) were added. The mixture was stirred for 4 h at 383K. Then water (200 mL) was added, which induced formation of yellow precipitate. The precipitate was filtered, washed with water (150 mL), dried and re-crystallized from ethanol. Yield 3.34 g (86%). Crystals suitable for X-ray analysis were obtained by slow evaporation of an ethanol solution.

### Refinement

H atoms were positioned geometrically, with C-H = 0.93, 0.97 and 0.96 Å for aromatic, methylene and methyl H, respectively, and constrained to ride on their parent atoms, with U_iso_(H) = xU_eq_(C,N), where x = 1.5 for methyl H and x = 1.2 for all other H atoms.  In the absence of significant anomalous dispersion effects, 1739 Friedel pairs were merged.

### Crystal data

**Table:**

C_21_H_22_N_2_O_4_	*D*_x_ = 1.319 Mg m^−^^3^
*M**_r_* = 366.41	Melting point: 421 K
Orthorhombic, *P**n**a*2_1_	Mo *K*α radiation, λ = 0.71073 Å
Hall symbol: P 2c -2n	Cell parameters from 25 reflections
*a* = 10.959 (2) Å	θ = 9–13°
*b* = 18.037 (4) Å	µ = 0.09 mm^−^^1^
*c* = 9.3330 (19) Å	*T* = 293 K
*V* = 1844.8 (6) Å^3^	Needle, brown
*Z* = 4	0.30 × 0.20 × 0.10 mm
*F*(000) = 776	

### Data collection

**Table:**

Enraf–Nonius CAD-4 diffractometer	1280 reflections with *I* > 2σ(*I*)
Radiation source: fine-focus sealed tube	*R*_int_ = 0.039
graphite	θ_max_ = 25.4°, θ_min_ = 2.2°
ω/2θ scans	*h* = 0→13
Absorption correction: ψ scan (North *et al.*, 1968)	*k* = −21→21
*T*_min_ = 0.973, *T*_max_ = 0.991	*l* = 0→11
3547 measured reflections	3 standard reflections every 200 reflections
1808 independent reflections	intensity decay: 1%

### Refinement

**Table:**

Refinement on *F*^2^	Primary atom site location: structure-invariant direct methods
Least-squares matrix: full	Secondary atom site location: difference Fourier map
*R*[*F*^2^ > 2σ(*F*^2^)] = 0.045	Hydrogen site location: inferred from neighbouring sites
*wR*(*F*^2^) = 0.115	H-atom parameters constrained
*S* = 1.00	*w* = 1/[σ^2^(*F*_o_^2^) + (0.060*P*)^2^] where *P* = (*F*_o_^2^ + 2*F*_c_^2^)/3
1808 reflections	(Δ/σ)_max_ < 0.001
244 parameters	Δρ_max_ = 0.21 e Å^−^^3^
2 restraints	Δρ_min_ = −0.13 e Å^−^^3^

### Special details

**Table:**

Geometry. All esds (except the esd in the dihedral angle between two l.s. planes) are estimated using the full covariance matrix. The cell esds are taken into account individually in the estimation of esds in distances, angles and torsion angles; correlations between esds in cell parameters are only used when they are defined by crystal symmetry. An approximate (isotropic) treatment of cell esds is used for estimating esds involving l.s. planes.
Refinement. Refinement of F^2^ against ALL reflections. The weighted R-factor wR and goodness of fit S are based on F^2^, conventional R-factors R are based on F, with F set to zero for negative F^2^. The threshold expression of F^2^ > 2sigma(F^2^) is used only for calculating R-factors(gt) etc. and is not relevant to the choice of reflections for refinement. R-factors based on F^2^ are statistically about twice as large as those based on F, and R- factors based on ALL data will be even larger.

### Fractional atomic coordinates and isotropic or equivalent isotropic displacement parameters (Å^2^)

**Table:**

	*x*	*y*	*z*	*U*_iso_*/*U*_eq_	
N1	0.4405 (3)	0.21405 (16)	0.7011 (4)	0.0561 (9)	
O1	0.2714 (3)	−0.10503 (17)	0.4672 (4)	0.0777 (10)	
C1	0.5640 (4)	0.2215 (2)	0.7609 (5)	0.0621 (11)	
H1A	0.6237	0.2174	0.6846	0.074*	
H1B	0.5788	0.1818	0.8288	0.074*	
N2	0.2974 (3)	−0.13210 (17)	0.7041 (4)	0.0553 (8)	
O2	0.3201 (3)	−0.15994 (17)	0.9379 (4)	0.0747 (9)	
C2	0.5780 (5)	0.2952 (2)	0.8348 (6)	0.0791 (15)	
H2A	0.6605	0.3000	0.8713	0.095*	
H2B	0.5223	0.2979	0.9154	0.095*	
O3	0.3649 (3)	−0.32513 (15)	0.7198 (5)	0.0858 (11)	
C3	0.5511 (5)	0.3589 (3)	0.7302 (7)	0.0898 (16)	
H3A	0.5523	0.4058	0.7811	0.108*	
H3B	0.6133	0.3604	0.6564	0.108*	
O4	0.4935 (3)	−0.22931 (16)	0.7031 (4)	0.0705 (8)	
C4	0.4263 (4)	0.3472 (2)	0.6620 (6)	0.0717 (13)	
H4A	0.3635	0.3527	0.7345	0.086*	
H4B	0.4129	0.3848	0.5894	0.086*	
C5	0.4158 (4)	0.2712 (2)	0.5944 (5)	0.0670 (12)	
H5A	0.3344	0.2645	0.5558	0.080*	
H5B	0.4737	0.2669	0.5161	0.080*	
C6	0.3992 (3)	0.1420 (2)	0.6730 (4)	0.0509 (10)	
C7	0.3691 (4)	0.1189 (2)	0.5352 (5)	0.0601 (11)	
H7A	0.3725	0.1524	0.4595	0.072*	
C8	0.3337 (4)	0.0458 (3)	0.5101 (5)	0.0618 (12)	
H8A	0.3136	0.0311	0.4175	0.074*	
C9	0.3280 (3)	−0.0048 (2)	0.6196 (5)	0.0515 (10)	
C10	0.3521 (3)	0.0175 (2)	0.7621 (4)	0.0461 (9)	
C11	0.3431 (3)	−0.0331 (2)	0.8754 (5)	0.0496 (10)	
C12	0.3581 (4)	−0.0101 (2)	1.0172 (5)	0.0586 (11)	
H12A	0.3535	−0.0441	1.0918	0.070*	
C13	0.3803 (4)	0.0646 (2)	1.0452 (5)	0.0557 (11)	
H13A	0.3860	0.0809	1.1395	0.067*	
C14	0.3935 (4)	0.1138 (2)	0.9362 (5)	0.0528 (10)	
H14A	0.4090	0.1633	0.9577	0.063*	
C15	0.3844 (3)	0.0918 (2)	0.7894 (4)	0.0460 (9)	
C16	0.2971 (4)	−0.0830 (2)	0.5885 (5)	0.0570 (11)	
C17	0.3207 (4)	−0.1124 (2)	0.8465 (5)	0.0538 (11)	
C18	0.2784 (3)	−0.21025 (19)	0.6780 (6)	0.0623 (12)	
H18A	0.2519	−0.2173	0.5797	0.075*	
H18B	0.2143	−0.2283	0.7405	0.075*	
C19	0.3916 (4)	−0.2541 (2)	0.7032 (5)	0.0586 (10)	
C20	0.4684 (6)	−0.3749 (3)	0.7462 (10)	0.131 (3)	
H20A	0.5438	−0.3514	0.7164	0.157*	
H20B	0.4742	−0.3861	0.8476	0.157*	
C21	0.4499 (6)	−0.4403 (3)	0.6680 (9)	0.145 (3)	
H21A	0.5188	−0.4726	0.6805	0.217*	
H21B	0.4409	−0.4285	0.5683	0.217*	
H21C	0.3775	−0.4647	0.7017	0.217*	

### Atomic displacement parameters (Å^2^)

**Table:**

	*U*^11^	*U*^22^	*U*^33^	*U*^12^	*U*^13^	*U*^23^
N1	0.059 (2)	0.0473 (17)	0.062 (2)	0.0060 (15)	−0.0043 (19)	0.0084 (18)
O1	0.086 (2)	0.076 (2)	0.071 (2)	0.0046 (17)	−0.0181 (18)	−0.025 (2)
C1	0.055 (3)	0.066 (2)	0.065 (3)	−0.0010 (19)	−0.005 (2)	0.016 (2)
N2	0.0492 (18)	0.0507 (18)	0.066 (2)	−0.0017 (14)	−0.0060 (18)	−0.0060 (19)
O2	0.094 (2)	0.0614 (18)	0.069 (2)	−0.0103 (17)	0.0184 (19)	0.0068 (18)
C2	0.087 (3)	0.068 (3)	0.082 (4)	−0.017 (2)	−0.024 (3)	0.014 (3)
O3	0.076 (2)	0.0521 (16)	0.129 (3)	0.0014 (15)	−0.010 (2)	−0.008 (2)
C3	0.115 (4)	0.060 (3)	0.095 (4)	−0.020 (3)	−0.017 (4)	0.020 (3)
O4	0.0456 (15)	0.0769 (19)	0.089 (2)	0.0008 (14)	0.0091 (17)	−0.0022 (19)
C4	0.089 (3)	0.053 (2)	0.073 (3)	0.005 (2)	0.000 (3)	0.004 (2)
C5	0.077 (3)	0.060 (3)	0.064 (3)	0.009 (2)	−0.007 (2)	0.014 (2)
C6	0.047 (2)	0.057 (2)	0.049 (3)	0.0132 (17)	−0.0027 (18)	0.005 (2)
C7	0.071 (3)	0.066 (3)	0.043 (2)	0.002 (2)	−0.008 (2)	0.001 (2)
C8	0.065 (3)	0.073 (3)	0.047 (3)	0.008 (2)	−0.012 (2)	−0.005 (2)
C9	0.044 (2)	0.056 (3)	0.055 (3)	0.0060 (18)	−0.0017 (18)	0.001 (2)
C10	0.0379 (19)	0.054 (2)	0.047 (2)	0.0054 (16)	−0.0007 (18)	−0.008 (2)
C11	0.048 (2)	0.050 (2)	0.051 (3)	−0.0010 (17)	0.006 (2)	0.002 (2)
C12	0.062 (3)	0.060 (3)	0.054 (3)	0.004 (2)	0.009 (2)	0.006 (2)
C13	0.065 (3)	0.063 (3)	0.039 (2)	0.000 (2)	−0.003 (2)	−0.001 (2)
C14	0.057 (3)	0.049 (2)	0.052 (2)	−0.0009 (19)	−0.005 (2)	−0.002 (2)
C15	0.045 (2)	0.049 (2)	0.044 (2)	0.0067 (16)	0.0014 (19)	−0.0080 (19)
C16	0.041 (2)	0.063 (3)	0.067 (3)	0.0070 (19)	−0.009 (2)	−0.014 (3)
C17	0.040 (2)	0.058 (3)	0.063 (3)	−0.0011 (18)	0.009 (2)	0.001 (2)
C18	0.047 (2)	0.050 (2)	0.090 (4)	−0.0024 (17)	0.002 (2)	−0.011 (2)
C19	0.056 (2)	0.054 (2)	0.066 (3)	0.0008 (19)	0.006 (2)	−0.007 (2)
C20	0.114 (5)	0.077 (3)	0.202 (9)	0.036 (3)	−0.058 (6)	−0.030 (5)
C21	0.138 (6)	0.103 (5)	0.193 (9)	0.036 (4)	−0.019 (6)	−0.014 (6)

### Geometric parameters (Å, °)

**Table:**

N1—C6	1.401 (5)	C6—C15	1.424 (6)
N1—C5	1.459 (5)	C7—C8	1.395 (6)
N1—C1	1.470 (5)	C7—H7A	0.9300
O1—C16	1.233 (5)	C8—C9	1.372 (6)
C1—C2	1.506 (6)	C8—H8A	0.9300
C1—H1A	0.9700	C9—C10	1.414 (6)
C1—H1B	0.9700	C9—C16	1.479 (6)
N2—C16	1.396 (6)	C10—C11	1.400 (5)
N2—C17	1.399 (6)	C10—C15	1.410 (5)
N2—C18	1.446 (4)	C11—C12	1.397 (6)
O2—C17	1.209 (5)	C11—C17	1.475 (5)
C2—C3	1.536 (7)	C12—C13	1.393 (6)
C2—H2A	0.9700	C12—H12A	0.9300
C2—H2B	0.9700	C13—C14	1.359 (6)
O3—C19	1.324 (5)	C13—H13A	0.9300
O3—C20	1.467 (6)	C14—C15	1.430 (6)
C3—C4	1.524 (7)	C14—H14A	0.9300
C3—H3A	0.9700	C18—C19	1.490 (6)
C3—H3B	0.9700	C18—H18A	0.9700
O4—C19	1.202 (5)	C18—H18B	0.9700
C4—C5	1.513 (6)	C20—C21	1.402 (7)
C4—H4A	0.9700	C20—H20A	0.9700
C4—H4B	0.9700	C20—H20B	0.9700
C5—H5A	0.9700	C21—H21A	0.9600
C5—H5B	0.9700	C21—H21B	0.9600
C6—C7	1.391 (6)	C21—H21C	0.9600
			
C6—N1—C5	117.9 (3)	C8—C9—C16	119.9 (4)
C6—N1—C1	116.9 (3)	C10—C9—C16	119.9 (4)
C5—N1—C1	111.5 (3)	C11—C10—C15	120.1 (3)
N1—C1—C2	110.4 (4)	C11—C10—C9	120.8 (3)
N1—C1—H1A	109.6	C15—C10—C9	119.1 (3)
C2—C1—H1A	109.6	C12—C11—C10	120.8 (4)
N1—C1—H1B	109.6	C12—C11—C17	118.8 (4)
C2—C1—H1B	109.6	C10—C11—C17	120.4 (4)
H1A—C1—H1B	108.1	C13—C12—C11	119.1 (4)
C16—N2—C17	125.0 (3)	C13—C12—H12A	120.4
C16—N2—C18	119.2 (4)	C11—C12—H12A	120.4
C17—N2—C18	115.7 (4)	C14—C13—C12	120.7 (4)
C1—C2—C3	110.4 (4)	C14—C13—H13A	119.7
C1—C2—H2A	109.6	C12—C13—H13A	119.7
C3—C2—H2A	109.6	C13—C14—C15	121.9 (4)
C1—C2—H2B	109.6	C13—C14—H14A	119.1
C3—C2—H2B	109.6	C15—C14—H14A	119.1
H2A—C2—H2B	108.1	C10—C15—C6	119.7 (4)
C19—O3—C20	116.2 (4)	C10—C15—C14	117.1 (4)
C4—C3—C2	109.6 (4)	C6—C15—C14	123.1 (4)
C4—C3—H3A	109.8	O1—C16—N2	120.4 (4)
C2—C3—H3A	109.8	O1—C16—C9	122.7 (4)
C4—C3—H3B	109.8	N2—C16—C9	116.9 (4)
C2—C3—H3B	109.8	O2—C17—N2	119.3 (4)
H3A—C3—H3B	108.2	O2—C17—C11	124.0 (4)
C5—C4—C3	111.6 (4)	N2—C17—C11	116.7 (4)
C5—C4—H4A	109.3	N2—C18—C19	111.7 (3)
C3—C4—H4A	109.3	N2—C18—H18A	109.3
C5—C4—H4B	109.3	C19—C18—H18A	109.3
C3—C4—H4B	109.3	N2—C18—H18B	109.3
H4A—C4—H4B	108.0	C19—C18—H18B	109.3
N1—C5—C4	110.0 (4)	H18A—C18—H18B	107.9
N1—C5—H5A	109.7	O4—C19—O3	124.4 (4)
C4—C5—H5A	109.7	O4—C19—C18	125.2 (4)
N1—C5—H5B	109.7	O3—C19—C18	110.3 (3)
C4—C5—H5B	109.7	C21—C20—O3	108.4 (5)
H5A—C5—H5B	108.2	C21—C20—H20A	110.0
C7—C6—N1	121.9 (4)	O3—C20—H20A	110.0
C7—C6—C15	119.2 (4)	C21—C20—H20B	110.0
N1—C6—C15	118.9 (4)	O3—C20—H20B	110.0
C6—C7—C8	120.3 (4)	H20A—C20—H20B	108.4
C6—C7—H7A	119.8	C20—C21—H21A	109.5
C8—C7—H7A	119.8	C20—C21—H21B	109.5
C9—C8—C7	121.1 (4)	H21A—C21—H21B	109.5
C9—C8—H8A	119.5	C20—C21—H21C	109.5
C7—C8—H8A	119.5	H21A—C21—H21C	109.5
C8—C9—C10	120.2 (4)	H21B—C21—H21C	109.5
			
C6—N1—C1—C2	−159.0 (4)	C11—C10—C15—C14	−6.7 (5)
C5—N1—C1—C2	61.3 (5)	C9—C10—C15—C14	172.7 (4)
N1—C1—C2—C3	−57.5 (5)	C7—C6—C15—C10	6.8 (5)
C1—C2—C3—C4	53.7 (6)	N1—C6—C15—C10	−175.1 (3)
C2—C3—C4—C5	−53.6 (6)	C7—C6—C15—C14	−169.4 (4)
C6—N1—C5—C4	160.5 (4)	N1—C6—C15—C14	8.8 (5)
C1—N1—C5—C4	−60.2 (5)	C13—C14—C15—C10	4.5 (5)
C3—C4—C5—N1	56.7 (5)	C13—C14—C15—C6	−179.3 (4)
C5—N1—C6—C7	18.9 (6)	C17—N2—C16—O1	−177.5 (4)
C1—N1—C6—C7	−118.2 (4)	C18—N2—C16—O1	5.7 (6)
C5—N1—C6—C15	−159.2 (4)	C17—N2—C16—C9	2.2 (6)
C1—N1—C6—C15	63.7 (5)	C18—N2—C16—C9	−174.6 (3)
N1—C6—C7—C8	177.0 (4)	C8—C9—C16—O1	−2.6 (6)
C15—C6—C7—C8	−4.9 (6)	C10—C9—C16—O1	177.6 (4)
C6—C7—C8—C9	−0.1 (6)	C8—C9—C16—N2	177.7 (4)
C7—C8—C9—C10	3.3 (6)	C10—C9—C16—N2	−2.1 (5)
C7—C8—C9—C16	−176.5 (4)	C16—N2—C17—O2	−179.0 (4)
C8—C9—C10—C11	178.1 (4)	C18—N2—C17—O2	−2.1 (5)
C16—C9—C10—C11	−2.1 (5)	C16—N2—C17—C11	1.8 (5)
C8—C9—C10—C15	−1.3 (6)	C18—N2—C17—C11	178.7 (3)
C16—C9—C10—C15	178.4 (3)	C12—C11—C17—O2	−3.6 (6)
C15—C10—C11—C12	4.1 (5)	C10—C11—C17—O2	174.7 (4)
C9—C10—C11—C12	−175.3 (4)	C12—C11—C17—N2	175.5 (4)
C15—C10—C11—C17	−174.2 (3)	C10—C11—C17—N2	−6.1 (5)
C9—C10—C11—C17	6.3 (5)	C16—N2—C18—C19	110.2 (4)
C10—C11—C12—C13	1.2 (6)	C17—N2—C18—C19	−66.9 (5)
C17—C11—C12—C13	179.5 (4)	C20—O3—C19—O4	2.9 (8)
C11—C12—C13—C14	−3.5 (6)	C20—O3—C19—C18	−179.7 (5)
C12—C13—C14—C15	0.6 (6)	N2—C18—C19—O4	−20.9 (7)
C11—C10—C15—C6	176.9 (3)	N2—C18—C19—O3	161.8 (4)
C9—C10—C15—C6	−3.7 (5)	C19—O3—C20—C21	−139.4 (6)

### Hydrogen-bond geometry (Å, °)

**Table:**

*D*—H···*A*	*D*—H	H···*A*	*D*···*A*	*D*—H···*A*
C1—H1A···O2^i^	0.97	2.60	3.455 (6)	147
C1—H1B···O1^ii^	0.97	2.51	3.373 (5)	149
C5—H5A···O2^iii^	0.97	2.44	3.219 (6)	138
C18—H18A···O1	0.97	2.29	2.735 (6)	107
C18—H18B···O4^iv^	0.97	2.56	3.315 (5)	135
C20—H20A···O4	0.97	2.27	2.671 (6)	103

Symmetry codes: (i) −*x*+1, −*y*, *z*−1/2; (ii) −*x*+1, −*y*, *z*+1/2; (iii) −*x*+1/2, *y*+1/2, *z*−1/2; (iv) *x*−1/2, −*y*−1/2, *z*.
